# 2181 Age-related change in 5-HT6 receptor availability in healthy male volunteers measured with 11C-GSK215083 PET

**DOI:** 10.1017/cts.2018.44

**Published:** 2018-11-21

**Authors:** Rajiv Radhakrishnan, Nabeel Nabulsi, Edward Gaiser, Jean-Dominique Gallezot, Shannan Henry, Beata Planeta, Shu-fei Lin, Jim Ropchan, Wendol Williams, Evan Morris, Deepak Cyril D’Souza, Yiyun Huang, Richard E. Carson, David Matuskey

**Affiliations:** Yale School of Medicine, Yale University, New Haven, CT, USA

## Abstract

OBJECTIVES/SPECIFIC AIMS: The serotonin receptor 6 (5-HT6) is a potential therapeutic target given its distribution in brain regions that are important in depression, anxiety, and cognition. This study sought to investigate the effects of age on 5-HT6 receptor availability using 11C GSK215083, a PET ligand with affinity for 5-HT6 in the striatum and 5-HT2A in the cortex. METHODS/STUDY POPULATION: In total, 28 healthy male subjects (age range: 23–52 years) were scanned with 11C-GSK215083 on the HR+PET scanner. Time-activity curves in regions-of-interest were fitted with multilinear analysis-1 method. Binding potentials (BPND) were calculated using cerebellum as the reference region and corrected for partial volume effects. RESULTS/ANTICIPATED RESULTS: In 5-HT6 rich areas, regional 11C-GSK215083 displayed a negative correlation between BPND and age in the caudate (*r*=−0.41, *p*=0.03) (14% change per decade), and putamen (*r*=−0.30, *p*=0.04) (11% change per decade), but not in the ventral striatum and pallidum. Negative correlation with age was also seen in cortical regions (*r*=−0.41, *p*=0.03) (7% change per decade), consistent with the literature on 5-HT2A availability. DISCUSSION/SIGNIFICANCE OF IMPACT: This is the first in vivo study in humans to examine the effect of age on 5-HT6 receptor availability. The study demonstrated a significant age-related decline in 5-HT6 availability (BPND) in the caudate and putamen.

